# Brazilian children’s quality of life during the COVID-19 pandemic: analysis of contextual factors and dimensions

**DOI:** 10.1590/1984-0462/2025/43/2023175

**Published:** 2024-09-23

**Authors:** Tainá Ribas Mélo, Luize Bueno de Araujo, Marcos Claudio Signorelli, Paulo Ricardo Bittencourt Guimarães, Vera Lúcia Israel

**Affiliations:** a Universidade Federal do Paraná, Matinhos, Curitiba, PR, Brazil.; b Centro Universitário de Brusque, Brusque, SC, Brazil.; c Faculdade Inspirar, Curitiba, PR, Brazil.

**Keywords:** Quality of life, Risk factors, Pandemics, COVID-19, Brazil, Children, Qualidade de vida, Fator de risco, Pandemia, COVID-19, Brasil, Criança

## Abstract

**Objective:**

The aim of this study was to investigate the contextual factors associated with the quality of life (QOL) of Brazilian children aged 0–12 years during the strict period of social isolation.

**Methods:**

This observational cross-sectional study was conducted between July and September 2020 using an online questionnaire on QOL-related family factors and the Pediatric Quality of Life Inventory (PedsQL™). Results were analyzed by multinomial logistic regression analysis.

**Results:**

The sample had 849 children, mostly from the South Region of Brazil (75%), white (83%), with typical development (79%), sedentary (68%), using screen (85%) for >3 h/day (44%). Their mothers were their main caregivers (90%). The following variables were significantly associated with high scores of QOL: typical health status (OR 2.38; 95%CI 1.60–3.55; screen time ≤2 h/day (OR 1.62; 95%CI 1.17–2.24); social distancing considered as “easy” (OR 1.67; 95%CI 1.20–2.32), and stimulation of the child by the family (OR 1.93; 95%CI 1.08–3.45).

**Conclusions:**

This study indicates that the family context can influence children’s QOL, especially during the COVID-19 pandemic and home environment reorganization.

## RESUMO

**Objetivo:**

Investigar os fatores contextuais associados à qualidade de vida (QV) de crianças brasileiras de zero a 12 anos, em momento de ápice de distanciamento social. Métodos: Estudo observacional, transversal, *online*, de julho a setembro de 2020, com questionário sobre fatores do contexto familiar associados à QV e Inventário Pediátrico sobre QV — PedsQL™. A análise dos dados foi feita por de regressão logística multinomial.

**Resultados:**

A amostra foi de 849 crianças, na maioria da Região Sul (75%), brancas (83%), com desenvolvimento típico (79%), sedentárias (68%), com uso de telas (85%) em tempo >3h/dia (44%). As mães eram as cuidadoras principais (90%). Foram significativamente associadas a escores mais elevados de QV: a condição de saúde típica (*odds ratio* — OR 2,38; intervalo de confiança de 95% — IC95% 1,60–3,55), o tempo de tela ≤2h/dia (OR 1,62; IC95% 1,17–2,24), o distanciamento social considerado “fácil” (OR 1,67; IC95% 1,20–2,32) e a família afirmar estimular a criança (OR 1,93; IC95% 1,08–3,45).

**Conclusões:**

Este estudo mostra que o contexto familiar pode influenciar a QV de crianças, especialmente no período de pandemia e de reorganização do ambiente domiciliar.

## INTRODUCTION

The COVID-19 pandemic began in Asia and quickly caused more than 5 million deaths worldwide.^1^ Many restriction measures were adopted to control the virus, particularly social distancing,^2^ leading to deep social changes.^3^ In Brazil, the first case was detected on February 26, 2020, and social distancing reached its peak (62.2%) in March 2020 — a critical moment of the pandemic,^4^ with many cases and deaths.^5^ Social distancing remained at 35–50% between April and September,^6^ the period when this research, the so-called #CRIANCAEMCASA. (#CHILDRENATHOME), was implemented.

Brazil is a continent-sized country, with great diversity and inequality,^7^ making it even more complex to manage the pandemic. Furthermore, there were no coordinated actions between the federal government and the states and municipalities, which took their own measures to minimize spreading of virus.^8^

Although children were the least affected by COVID-19, they were likewise impacted by the pandemic.^9^ Health services were changed and interrupted, classes were canceled and replaced with remote teaching, social interaction with peers was restricted, and families started working from home, lost their jobs, and/or faced financial and health difficulties,^10^ resulting in a critical development phase for children due to their neuroplasticity.^2^ Possible health risks include both the virus itself^11^ and the pandemic from a broader biopsychosocial (BPS) perspective, which considers social and psychological factors as well.^12^ The health status can be verified through quality-of-life (QOL) indicators^13^ regarding functions and structures, activities and participation, and contextual factors, according to the International Classification of Functioning, Disability, and Health (ICF),^14^ and related social determinants.^3^

Considering the social transformations and restrictions imposed by the pandemic, this research aimed to investigate the greatest risk and protection contextual factors associated with 0- to 12-year-old Brazilian children’s QOL, following a BPS model, at the peak social distancing during the COVID-19 pandemic.

## METHOD

The research, called #CRIANCAEMCASA (#CHILDRENATHOME), followed human research ethics according to Resolution 466/12 of the National Health Council (Brazil) and was approved by the Research Ethics Committee of Federal University of Paraná under CAAE no. 32679520.4.0000.0102 (evaluation report no. 4.146.615).

The research followed the recommendations in Strengthening the Reporting of Observational Studies in Epidemiology (STROBE Statement)^15^ for cross-sectional design and convenience samples. The assessment instruments were systematized according to the ICF BPS model^14^ to investigate QOL outcomes and associated risk and protective contextual factors. The assessment instruments, questionnaire, and Pediatric Quality of Life Inventory (PedsQL™) were standardized regarding health status in functioning dimensions (functions, structures, activities, and participation) and contextual factors (environmental and personal).

The research was publicized through social media (Instagram and Facebook), WhatsApp, and e-mails to caregivers of 0- to 12-year-old Brazilian children of both sexes residing in Brazil. The sampling approach used for this research was the stratified random sampling plan, with strata defined by age group and Brazilian state, with an acceptable 5% margin of error and 95% confidence level, which indicated the need for 385 responses nationwide.

Research forms included a questionnaire developed by the researchers with general questions on identification, the region in Brazil, sex, race, age, respondents’ data (children’s caregivers), children’s health condition (typical/healthy, respiratory diseases, disabilities, COVID-19), healthcare (public, private, or both), home environment (type of housing, basic sanitation/sewage, water supply, paved street), socioeconomic (family income, government welfare, children’s main caregiver’s/parents’ employability), educational situation (grade in school, daycare/school attendance), stimulation received by family (games or educational activities carried out with the child), media use (screen time and type), life habits (physical activity), and social distancing during the COVID-19 pandemic (binary response: easy or difficult). They were used in all age groups to determine risk or preventive factors associated with QOL.

PedsQL™ was used online with permission of the Mapi Research Trust (http://www.mapi-trust.org) to analyze QOL outcomes. It is validated for both interview and online use^16^ and reliable,^17^ in the versions for 1- to 12-month- and 13- to 24-month-old babies^17^ and 2- to 12-year-old children. The PedsQL™ (Version 4.0 Short Form (SF15) — Portuguese [Brazil]) addresses toddlers (2–4 years old),^18^ small children (5–7 years old), and children (8–12 years old).^18^ All questionnaires are of low cost and take about 5 min to answer;^18^ they were made available online to the children’s caregivers, who were instructed to answer the questions considering the previous month. The instruments assess QOL with age-specific questions in the following dimensions: physical functioning (PF), emotional functioning (EF), social functioning (SF), and cognitive functioning (CF) or school functioning (ScF), with a total score. Physical symptoms (PS) are an additional dimension for children under 2 years old. ScF questions accepted home tasks and activities, given the social distancing imposed by the pandemic.

The answers follow a five-level Likert scale^16^ for conditions and/or problems: (0=never; 1=almost never; 2=sometimes; 3=often; and 4=almost always). After being scored, the items are linearly transposed to an inverted scale from 0 to 100 (0=100, 1=75, 2=50, 3=25, and 4=0) to calculate each dimension’s mean scores.^18^ Then, the physical health summary (PhyHS — encompassing PF [for all ages] and PS [for babies]) and psychosocial health summary (PsyHS — calculated with the mean of EF, SF, and CF or ScF) were calculated.^19^

PedsQL™ for 1- to 12-month-old babies included this age group and those under 1 month old. In data analysis, CF/ScF were considered equivalent to compare dimensions and establish the sample’s total score, encompassing all age groups. The age groups were organized as follows: 0–12 months; 13–23 months; 2–4 years; 5–7 years; 8–12 years; and all ages together (0–12 years). The total score was obtained from the mean of the PhyHS and PsyHS. PedsQL™ does not have a normative 0 to 100 QOL score — higher scores indicate better QOL.^18^

First, a study pilot was made for adjustments, resulting in specific forms for each age group, made available in Linktree. The researchers publicized this research through social media (Instagram, Facebook) in a profile created specifically for it, and through WhatsApp to other researchers and possible known participants that might help publicize it to others; hence, the participants helped pass on the link to the research (snowball) to other potential participants. Moreover, the research was shared via available institutional e-mail to health researchers and program coordinators nationwide.

The link to the research first led to an online informed consent form. Those who agreed to participate answered the questionnaire in Google Forms between July and September 2020.

Regarding QOL and risk/protective factors, the sample was classified into three groups, based on PedsQL™ total score for all ages and z-score, namely, 0=sample’s mean score ±1 standard deviation (SD) (medium); +1=above reference (high); and -1=below reference (low). One SD in relation to the sample has already been used as a reference cutoff in this scale.^20^

Sociodemographic and environmental variables were organized, grouped into two categories, and analyzed concerning the outcome variable (QOL/PedsQL™) with a multinomial logistic regression model in Statistica^®^, version 7. Variables scoring >90% in a single category were excluded from the model due to their low discriminatory potential. The relationship between these variables was measured by chi-square test, and significant variables were included in the model, considering only the main effects. There was no interaction with independent variables, given the loss of sample power because of the complex interactions. No multicollinearity problem for the adjustments was identified. Odds ratios were calculated to estimate the associated values between variables of interest and the outcome variable (QOL).

## RESULTS

The survey forms were accessed, through Linktree control, approximately 5000 times and, excluding repeated responses, a total of 849 valid responses, with a response rate of 17%, were recorded.

**Figure 1:**
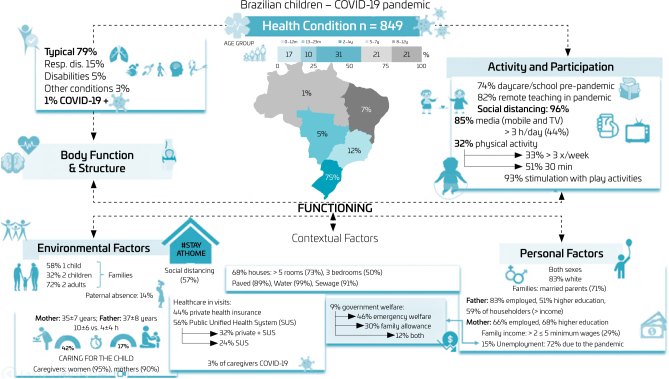
Health condition, activity and participation, and contextual factors of Brazilian children (0–12 years old) during the Covid-19 pandemic (July-September 2020).

Figure 1 shows data on the children’s health status, activity and participation, and personal and environmental contextual factors related to the investigated children (n=849) and on the caregivers’ main characteristics. Tables 1 and 2 describe, per age group and all ages combined, variables in the regression model (Tables 3 and 4) significant to identify risk/protective factors and/or relevant to explain the findings.

Resp. dis: respiratory diseases; m: months; y: years; h: hours; SUS: *Sistema Único de Saúde.*

**Table 1:** Activity and participation and contextual factors in Brazilian children (0–12 years old) during the COVID-19 pandemic (n=849).

	Age group (months/years)					
	0–12 months	13–23 months	2–4 years	5–7 years	8–12 years	0–12 years
n	139	85	266	180	179	849
	n (%)					
Media use	42 (30.2)	63 (64.1)	258 (97.0)	180 (100)	177 (98.9)	184 (59.9)
Type of screen*						
Mobile phone	15 (35.7)	29 (46.0)	169 (65.5)	153 (85.0)	158 (89.3)	90 (48.9)
Tablet	4 (9.5)	10 (15.8)	88 (34.1)	71 (39.4)	57 (32.2)	27 (14.7)
Computer	0 (0.0)	6 (9.5)	31 (12.0)	66 (36.7)	124 (70.0)	13 (7.1)
Television	38 (90.5)	62 (98.4)	238 (92.3)	176 (97.8)	158 (89.3)	169 (91.8)
Videogame	0 (0.0)	0 (0.0)	17 (6.6)	48 (26.7)	75 (42.4)	1 (0.5)
Screen time* ^,†^						
Up to 30 minutes	14 (33.3)	5 (7.9)	8 (3.1)	3 (1.7)	2 (1.1)	22 (12.0)
Up to 1 hour/day	18 (28.6)	19 (30.2)	35 (13.6)	18 (10.0)	5 (2.8)	41 (22.3)
Up to 2 hour/day	11 (26.2)	13 (20.6)	53 (20.6)	25 (13.9)	21 (11.9)	44 (23.9)
Up to 3 hour/day	4 (9.5)	16 (25.4)	75 (29.1)	45 (25.0)	20 (11.3)	43 (23.4)
>3 hour/day	1 (2.4)	10 (15.9)	87 (33.7)	89 (49.4)	129 (72.9)	34 (18.5)
Stimulation-play^†^	137 (98.6)	84 (98.8)	257 (96.6)	168 (93.3)	145 (81.0)	303 (98.7)
Paternal absence^†^	18 (13.0)	10 (11.8)	26 (9.8)	31 (17.2)	36 (20.1)	121 (14.3)
Time fathers spent with the children (hour/day)	4±3	4±4	4±3	4±4	4±4	4±4
Attended daycare/school^†^ before the pandemic	6 (4.3)	39 (45.9)	227 (85.3)	179 (99.5)	175 (97.8)	626 (73.7)
Typical health status^†^	125 (89.9)	71 (83.5)	216 (81.2)	134 (74.4)	125 (69.8)	669 (78.8)
Social distancing-easy^†^	31 (22.3)	15 (17.7)	56 (21.1)	42 (23.3)	50 (27.9)	194 (22.9)

*Percentages calculated based on the number of children who used media/were physically active;

^†^Significant variables to the regression model.

**Table 2:** Physical activity in Brazilian children (0–12 years old) during the COVID-19 pandemic (n=849).

	Age group (months/years)					
	0–12 months	13–23 months	2–4 years	5–7 years	8–12 years	0–12 years
n	139	85	266	180	179	849
	n (%)					
Physically active	5 (3.6)	10 (11.8)	76 (28.6)	89 (49.4)	87 (48.6)	30 (9.8)
Frequency of PA* (x/week)						
1	8 (80.0)	1 (10.0)	15 (19.7)	10 (11.2)	8 (9.2)	10 (33.3)
2	1 (20.0)	1 (10.0)	17 (22.4)	27 (30.3)	33 (37.9)	6 (20.0)
3	0 (0.0)	2 (20.0)	12 (15.8)	22 (24.7)	26 (29.9)	2 (6.7)
>3	0 (0.0)	6 (60.0)	31 (42.1)	30 (33.7)	20 (23.0)	12 (40.0)
Duration of PA^b^						
30 minutes	5 (100)	6 (60.0)	44 (57.9)	45 (50.6)	36 (41.4)	20 (66.7)
1 hours	0 (0.0)	3 (30.0)	14 (18.4)	34 (38.2)	33 (37.9)	6 (20.0)
>1 hours	0 (0.0)	1 (10.0)	18 (23.7)	10 (11.2)	18 (20.7)	4 (13.0)

PA: physical activity. Significant variables to the regression model.

*Percentages calculated based on the number of children who used media/were physically active.

**Table 3:** Variables of the regression model associated with the quality of life — PedsQL™ (n=849).

Age group	Associated variables	Effect level	PedsQL™ score response level	Estimate	Standard error	Wald	p	OR	LL	UL
0–12 years	Paternal absence	No	High	0.44	0.22	3.92	0.05	1.56	1.00	2.42
	Health condition	Typical	High	0.87	0.20	18.11	**<0.01**	2.38	1.60	3.55
	Screen time	≤2 h/day	High	0.48	0.16	8.59	**<0.01**	1.62	1.17	2.24
	Stimulation at home	Yes	High	0.66	0.30	4.96	**0.03**	1.93	1.08	3.45
	Social distancing	Easy	High	0.51	0.17	9.26	**<0.01**	1.67	1.20	2.32
	Time fathers spent with the children	<3±3 h/day	Medium	-0.33	0.12	7.39	**0.01**	0.72	0.57	0.91
	Paternal absence	No	Medium	0.34	0.15	5.19	**0.02**	1.41	1.05	1.89
	Health condition	Typical	Medium	0.38	0.12	10.68	**<0.01**	1.46	1.16	1.84
	Stimulation at home	Yes	Medium	0.52	0.17	9.44	**<0.01**	1.68	1.21	2.34

h: hours; OR: odds ratio; LL: lower limit; UL: upper limit. In bold: p<0.05 (multinomial logistic regression).

**Table 4:** Variables of the regression model associated with the quality of life — PedsQL™ per age range (n=849).

Age group	Associated variables	Effect level	PedsQL™ score response level	Estimate	Standard error	Wald	p	OR	LL	UL
0–12 m	Attended daycare/school	No	High	–8.11	0.49	275.61	**<0.01**	0.0003	0.0001	0.0008
	Screen time	≤2 h/day	High	–7.72	0.60	167.46	**<0.01**	0.0004	0.0001	0.0014
13–23 m	Householder	Father	High	1.76	0.34	26.78	**<0.01**	5.80	2.98	11.30
	Paternal age	>37±8 years	High	–10.71	0.48	490.74	**<0.01**	0.00002	0.00001	0.00006
	Time fathers spent with the children	<3±3 h/day	High	1.37	0.35	14.86	**<0.01**	3.92	1.96	7.86
	Maternal age	<37±7 years	High	–9.82	0.48	425.35	**<0.01**	0.00005	0.00002	0.00014
	Paternal absence	No	High	6.06	0.74	66.32	**<0.01**	430.31	99.97	1852.11
	Health condition	Typical	High	5.64	0.58	94.65	**<0.01**	282.41	90.60	880.32
	Attended daycare	No	High	3.96	0.32	150.72	**<0.01**	52.52	27.90	98.85
	Social distancing	Easy	High	1.99	0.48	16.92	**<0.01**	7.30	2.83	18.83
	Stimulation at home	Yes	Medium	2.15	837.19	0.00	**<0.01**	8.60	0.00	-
2–4 y	Health condition	Typical	High	1.09	0.41	6.90	**0.01**	2.97	1.32	6.69
	Screen time	≤2 h/day	High	7.34	0.49	220.48	**<0.01**	1534.77	582.77	4041.90
	Time fathers spent with the children	<3±3 h/day	Medium	–0.78	0.35	4.92	**0.03**	0.46	0.23	0.91
	Stimulation at home	Yes	Medium	1.09	0.49	5.05	**0.02**	2.98	1.15	7.72
5–7 y	Screen time	≤2 h/day	High	0.89	0.41	4.61	**0.03**	2.43	1.08	5.47
	Social distancing	Easy	High	0.91	0.39	5.28	**0.02**	2.47	1.14	5.35
	Health condition	Typical	Medium	0.46	0.22	4.30	**0.04**	1.58	1.03	2.43
8–12 y	Health condition	Typical	High	1.58	0.82	3.74	0.05	4.86	0.98	24.08
	Attended daycare/school	No	High	1.75	0.90	3.78	0.05	5.76	0.98	33.69
	Social distancing	Easy	Medium	0.55	0.26	4.60	**0.03**	1.73	1.05	2.85

m: months; y: years; h: hours; OR: odds ratio; LL: lower limit; UL: upper limit. In bold: p<0.05 (multinomial logistic regression).

Responses encompassed all age groups and the five regions of the country (South: 75%). Most children were white (83%) and typically healthy (79%). Most healthcare was from the public Unified Health System (SUS, in Portuguese) (56%). Positive COVID-19 results were reported by 3% of respondents and 1% of children. Almost 74% of them attended school/daycare before the pandemic, during which most (82%) were on remote teaching. Most caregivers (57%) and children (96%) stayed home during the research period, which 73% of the sample considered difficult.

Most children lived in a house (68%), with sewage (91%), piped water or well (99%), and on a paved road (89%). Most caregivers (93%) stimulated the children with play activities at home — more frequently in those under 5 years old. Most caregivers were women (95%), young (35±7 years), married (71%), and the children’s mothers (90%); they spent approximately 43% of their daily time (10±6 h/day) — in contrast with the fathers’ 17% (4±4 h/day) — caring for the children. Most were only children (58%) or lived with another child at home (32%), in families having two adults (72%). In most families, the father was the householder, i.e., had the highest income (59%). Paternal absence was reported in 14% of families — more frequently when children were older than 5 years (Figure 1; Table 1).

Fathers (37±8 years) were unemployed in 17% and mothers (35±7 years) in 34% of the cases. Most fathers and mothers had a higher education degree (51% and 68%). The caregivers’ mean income was 2 to 5 minimum wages (MW) (29%), ≤2 MW (25%), and 5 to 10 MW (24%). Unemployment was reported by 15% of caregivers — 72% due to the pandemic. Only 9% of families received welfare — emergency pandemic welfare (46%).

Tables 2 and 3 show the details of important variables in the regression model, associated with QOL per age group. It also presents variables that, though not identified through the model (media use, type of media, physical activities), showed evidence (Lin et al., 2020) of relationships with sedentarism and functioning.

Most children routinely used media at home (85%) — mostly mobile phones (73%) and television (66%) — for more than 3 h/day (44%). In the observation per age range, the older the children, the more they used different media and for longer (Table 1).

Concerning physical activity (Table 2), most children (68%) were sedentary (0–12 years); children above 5 years old were physically active more often. As for those who were physically active (32%), most of them (51%) were so for 30 minutes, more than three times a week (33%).

**Figure 2:**
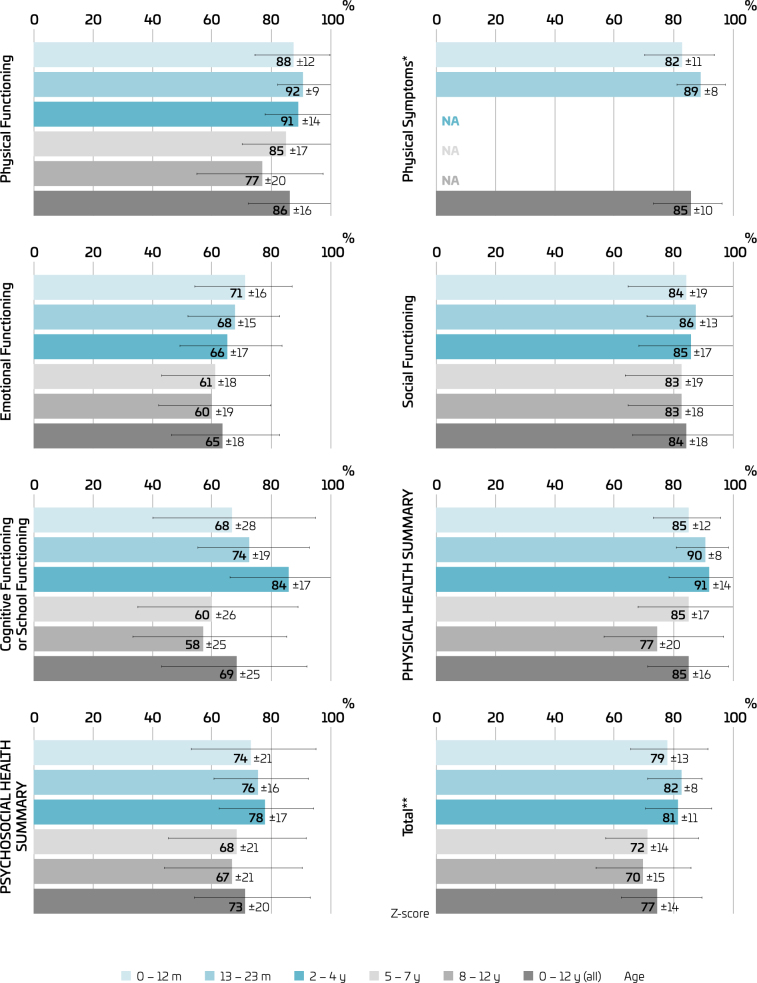
Quality of life of Brazilian children (n=849) during the COVID-19 pandemic (July–September 2020).

NA: Not applicable; m: months; y: years.

PedsQL™ analysis shows (Figure 2) higher PhyHS (85±16) than PsyHS (73±20) in the whole sample and per age group. The total score for 0–12 years was 77±14, and the mean (reference) z-score was 62.96 at 90.1% — corresponding to 69% of the sample, while 16% had a low score.

*Up to 23 months only; **Average value of physical and psychosocial health summaries.

Dimensional descriptive analysis showed lower ScF values for 5–7 years and 8–12 years (60±26; 58±25) and EF values for 5–7 years and 8–12 years (61±18; 60±19). Children aged 5–7 years and 8–12 years had the worst total QOL scores (72±14; 70±15). Scores were higher in PF (>80) in all ages, except for 8–12 years (77±20). Children aged 8–12 years, followed by 5–7 years, had lower QOL scores than the others. Despite the social distancing, the SF dimension had the most similar scores between age groups and in the mean of all age groups.

The following effect-level variables were included in the 0–12-year multinominal logistic regression analysis: householder (father), paternal age (>37±8), time fathers spent with children (<3±3 h/day), maternal age (<37±7), paternal absence (no), health status (healthy), daycare attendance (no), screen time (<2 h/day), family stimulation at home (yes), and social distancing (easy). “High” and “medium” QOL responses were analyzed in contrast with “low” ones.

The following variables were significantly associated with QOL of 0- to 12-year-old (Table 3) children with a “high” response level and possible protection factors: children with a typical health status, by 2.38 times (ß=0.87; p<0.01; 95%CI 1.60–3.55); screen time ≤2 h/day, by 1.62 times (ß=0.48; p<0.01; 95%CI 1.17–2.24); social distancing considered “easy” by caregivers, by 1.67 times (ß=0.51; p<0.01; 95%CI 1.20–2.32); and children stimulated by caregivers, by 1.93 times (ß=0.66; p=0.03; 95%CI 1.08–3.45). As for “medium” QOL response level, the following were significantly associated: typical health status, by 1.46 times (ß=0.38; p<0.01; 95%CI 1.16–1.84); children being stimulated, by 1.68 times (ß=0.52; p<0.01; 95%CI 1.21–2.34); and non-absent father, by 1.41 times (ß=0.34; p=0.02; 95%CI 1.05–1.89). The less time fathers spent caring for the children (<3±3 h/day) (ß=-0.33; p=0.01; 95%CI 0.57–0.91) was identified as a risk factor.

Significant associations with the outcome and the estimated odds ratios (OR) per age range are presented below. In those aged 0–12 months, no variables were associated with the outcomes, given the almost null OR values.

For 13–23 months: father as householder (ß=1.76; p<0.01; OR 5.80; 95%CI 2.98–11.30), time fathers spent with children <3±3 h/day (ß=1.37; p<0.01; OR 3.92; 95%CI 1.96–7.86), non-absent father (ß=6.06; p<0.01; OR 430.31; 95%CI 99.97–1852.11), typically healthy (ß=5.64; p<0.01; OR 282.41; 95%CI 90.60–880.32), daycare nonattendance (ß=3.96; p<0.01; OR 52.52; 95%CI 27.90–98.85), and caregivers’ “easy” social distancing (ß=1.99; p<0.01; OR 7.30; 95%CI 2.83–18.83).

For those 2–4 years old, typically healthy (ß=1.09; p=0.01; OR 2.97; 95%CI 1.32–6.69) and screen time ≤2 h/day (ß=7.34; p=0.03; OR 1534.77; 95%CI 582.77–4041.90) were protective factors for “high” QOL scores. In contrast, the time fathers spent with children <3±3 h/day (ß=-0.78; p=0.03; OR 0.46; 95%CI 0.23–0.91) was a risk factor for “medium” QOL score, while “easy” social distancing was a protective factor (ß=1.09; p=0.02; OR 2.98; 95%CI 1.15–7.72).

For those 5–7 years old, screen time ≤2 h/day (ß=0.89; p=0.03; OR 2.43; 95%CI 1.08–5.47) and “easy” social distancing (ß=0.91; p=0.02; OR 2.47; 95%CI 1.14–5.35) were associated with “high” QOL scores while being typically healthy was associated (ß=0.46; p=0.04; OR 1.58; 95%CI 1.03–2.43) with “medium” QOL score.

For those 8–12 years old, even though “easy” social distancing was associated with a “medium” QOL score (ß=0.55; p=0.03; OR 4.86; 95%CI 0.98–24.08), the confidence intervals indicate that this finding is inconclusive.

## DISCUSSION

Based on the total reference score and QOL categories, 16% of the children had a “low” score. Contextual factors were analyzed for the whole sample (0–12 years) and per age group.

In PedsQL™ analysis, PhyHS (85±16) was higher than PsyHS (73±20) in 0- to 12-year-old children and each age group. The scale validation process identified similar PhyHS and PsyHS values in 2–16 years (84±20 and 81±15, respectively).^20^ The discussion considered each PhyHS and PsyHS dimensions and the total score used to analyze the associated contextual factors.

The sample’s total QOL score was 77±14, and the reference interval was 62.96 at 90.1%, considering ±1 SD. This interval encompasses values found in 4- to 18-month-old babies from Curitiba (Paraná, Brazil) (76.9±9),^21^ in typical 5–7 and 8–12-year-old Portuguese children (68.8±17.7 and 75.9±15.2),^22^ healthy 2–18-year-old children from São Paulo (Brazil) (88.9±7.4),^18^ and healthy children from various countries — the USA, Canada, the Netherlands, and Bosnia and Herzegovina (ranging from 80 to 89)^23^ — before the pandemic. Hence, in our study, Brazilian children’s QOL during COVID-19 social isolation was like that observed before the pandemic.

Age group comparisons also revealed that children aged 5–7 years and 8–12 years had the worst total QOL scores (72±14; 70±15), suggesting that the pandemic impacted more of this age range’s QOL. Scores were higher in PF (>80) in all ages—except for 8–12 years (77±20), which are near those found in a previous study^18^ in 2- to 18-year-old Brazilian children with pathologies (76±22.7), while healthy children had higher scores (95.9±5.8). However, this validation study did not report each age group’s specific values, limiting comparisons with the present study.

Lower ScF and EF values in 5–7 and 8–12 years are like the main values found in Portuguese children before the pandemic.^22^

Interestingly, 8- to 12-year-old children had lower QOL scores than others—also below the reference QOL mean (80.9) suggested by Ow and Mayo^13^ in a study that verified values for children older than 5 years from various countries. However, the values were similar to those of a 2014 study^22^ on Portuguese children. These divergences reinforce the need for populational investigations addressing each country’s social needs to compare and establish reference scores.

Furthermore, puberty sets differences in well-being, as QOL is influenced by sex and age.^13^ This was also found in a study conducted in Indonesia.^2^ Hence, Brazilian results indicate that 5–7 and 8–12-year-old children — age groups with intense social and school relationships — have lower QOL scores. Although sex differences were not comparatively analyzed in the present study, sex was not a significant variable in this model.

ScF and EF values were similar to mean scores found in Brazilian children in 2014,^22^ although EF scores were lower than in other QOL dimensions. Previous study^24^ identified anxiety and depression in Brazilian children aged 6 to almost 12 years, possibly because of stress from the pandemic and the fewer physical activity opportunities. Being less physically active has been previously reported as a predisposing factor to emotional problems.^25^ Physical activities could help minimize these problems,^26^ especially in children with scores below the mean — “low” scores in the present study.

Lower ScF scores in 5–7 and 8–12 years make sense, as in-person classes were canceled and replaced with remote teaching. This may point to future learning difficulties, particularly in children with lower scores and therefore at greater risk.

Sedentarism was identified in children isolated at home, which, in combination with media use and high screen time, resulted in their low percentages of physical activity. Most children used media, especially mobile phones, and television, for more than 3 h/day, which agrees with reports in other studies.^27^ All groups used media, progressively increasing with age. Despite the screen time in school-age children (which may be ascribed to school activities), daily usage time (>3 h) is greater than those recommended by the Brazilian Society of Pediatrics^28^ — for all ages (<2 years: no use; 2–5 years: up to 1 h/day; 6–10 years: 1–2 h/day; ≥11 years: 2–3 h/day).

These results agree with another study on Brazilian children under 13 years old (March to April 2020), which identified that staying at home during the pandemic reduced physical activity by 46% and increased screen use by 38%.^27^ This is similar to what has been found in Brazilian adults,^29^ and Indonesian^2^ and Chinese children and adolescents.^30^ Considering the limited interaction with other children — particularly as most families had only one child at home—greater contact with adults may explain such sedentarism and greater screen time.

Although the pandemic increased family activities, some studies^24,31^ identified that playing without physical activity predominated, often accompanied by media use.

The relationship between less screen time and better QOL outcomes calls attention to overall consequences for children’s health and well-being. Evidence indicates that excessive use may have emotional and/or behavioral consequences for children.^32^ Given the increasing media use at all ages,^33^ this situation calls for attentive follow-up. Besides screen time, the quality of use must be further investigated to provide more precise guidance on specific media use per age group and its risks and/or benefits.

Thus, screen time must also be addressed concerning SF and physical activity. Less screen time may enable greater social interaction, even when limited to the family — which was a protective factor for children’s QOL in this study. Inversely, high screen time tends to diminish active play, impairing children’s development and well-being.

The typical health status may be related to caregivers’ overall perception of children’s well-being, which encompasses various QOL dimensions. Better QOL scores were found in typically developed babies than in those at risk/delay^21,34^ — and higher scores reflect a better QOL,^16^ as there are fewer changes in health status.

Interestingly, caregivers’ “easy” social distancing — which occurred in few (21.9%) families — was a protective factor for children’s QOL.

Research children’s main caregivers were women, young, married, and their mothers, who took 40% of their daily time — 2.8 times greater than the fathers’ — caring for the children. Mothers’ greater participation in research on children’s health^35,36^ confirms their role as the main caregiver. The less time fathers spent with children was a risk factor for 0–12 and 2–4-year-old children’s QOL, as well as paternal absence in 0–12 years and 13–23 months old.

These data corroborate both the women’s being the greatest workforce in childcare and household chores^37^ and the increased risk to children due to paternal absence.^38^

Not attending a daycare center was a protective factor for those aged 13–23 months. These data must be cautiously interpreted, as only 4.5% of the 0- to 12-month-old children attended daycare before the pandemic, which may justify the almost null OR values; while 45% of the 13- to 23-month-old ones did so — and during the pandemic, these babies remained at home. However, the literature reports both negative^39^ and positive daycare influences^40^ on small children who attend daycare centers.

Protective social interaction factors for QOL corroborates health analysis in the ICF BPS model. It addresses environmental influences,^41^ including parental relationships and interactions,^41^ agreeing with QOL multidimensionality. These factors had to be adjusted during social distancing, which may have long-term consequences for children’s neuropsychomotor development, as the quality of the environment the children could explore was limited^42^ and the exposure to situations of risk and violence increased.^43^

Despite the high screen use and less physical activity, most families stimulated children at home, which was a protective factor for better QOL scores (0–12 years), recognizing the positive effects of parental stimulation.^44^ Stimulation opportunities at home are known to be associated with neuropsychomotor development, which may influence the QOL.^45^

Remote teaching during the pandemic may have failed to reach more vulnerable populations. QOL is multidimensional, and the three Southern states — the most represented in this study — have the lowest inequality rates in the Gini index (Santa Catarina, Paraná, and Rio Grande do Sul),^7^ the highest human development indices,^46^ and the lowest incidence and mortality rates from COVID-19 in 2020.^7^ Hence, worse and more complex situations can be expected in more vulnerable populations, as Brazil has great inequalities,^7,46^ including in access to media and the Internet. In 2018, about 10–16% of schoolchildren still had no access to the Internet, with differences between the states and less access in poorer ones.^47^

Thus, this study’s contextual factors associated with children’s QOL may not represent Brazil as a whole. Nonetheless, considering QOL as a health indicator for Brazilian children during the pandemic, the differential of this research is the BPS analysis, with validated, quick, low-cost instruments^21,48^ — which may identify associated factors faster to plan future measures on both the individual (children in their families) and macro levels (pediatric healthcare management), having a multiprofessional team use family-centered intervention programs^49^ and instruments such as PedsQL^®^.

Some limitations of the study need to be pointed out. The results should not be extrapolated to the entire Brazilian population, considering that, because of the sample size and due to the distancing measures and the online nature of the survey, the respondents do not represent the entire diversity of the country. Furthermore, those interested in the research may possibly be the ones with the greatest interest and even the most experience in childcare. With regard to the territory, because Brazil is a continental country, with vast geographical, social, and economic diversity, even in the pandemic, the COVID-19 control measures varied greatly among the different regions of Brazil, limiting generalizations. Also, the QOL, as stated by PedsQL™, reflects the perception of caregivers (and not children’s perception), depending on their perspective and commitment to a true response possible.

The present study identified that, during a period of social distancing due to the COVID-19 pandemic, the protective factors associated with Brazilian children’s QOL were the typical health condition, less exposure to screens (screen time ≤2 h/day), social distancing considered easy” by families, and receiving stimulus (games or educational activities carried out with the child) from the family at home.

## Data Availability

The database that originated the article is available with the corresponding author.
